# Supplementary material for: HLTF disrupts Cas9-DNA post-cleavage complexes to allow DNA break processing
Source: Nat Commun. 2024 Jul 10;15:5789. doi: 10.1038/s41467-024-50080-y (PMC11237066; doi:10.1038/s41467-024-50080-y)
Supplement: Supplementary file 1 — Supplementary Information [file 41467_2024_50080_MOESM1_ESM.pdf]

Supplementary Information

HLTF disrupts Cas9-DNA post-cleavage complexes to allow DNA break processing

Giordano Reginato, Maria Rosaria Dello Stritto, Yanbo Wang, Jingzhou Hao, Raphael Pavani, Michael Schmitz, Swagata Halder, Vincent Morin, Elda Cannavo, Ilaria Ceppi, Stefan Braunshier, Ananya Acharya, Virginie Ropars, Jean-Baptiste Charbonnier, Martin Jinek, Andre Nussenzweig, Taekjip Ha, and Petr Cejka

Table of content:

Supplementary Figures 1-7

Supplementary Table 1: Oligonucleotides used in this study.

Supplementary Table 2: Synthetic genes sequence.

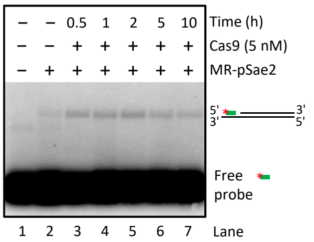

Supplementary Figure 1. Cas9-dependent breaks are invisible to DNA end resection enzymes, related to Figure 1.

Representative annealing DNA end resection assay of plasmid-based substrate incubated with Cas9 for the indicated time before addition of the resection components (25 nM Mre11-Rad50 with 200 nM phosphorylated Sae2, MR-pSae2). A representative of three independent experiments is shown.

Source data are provided as a Source Data file.

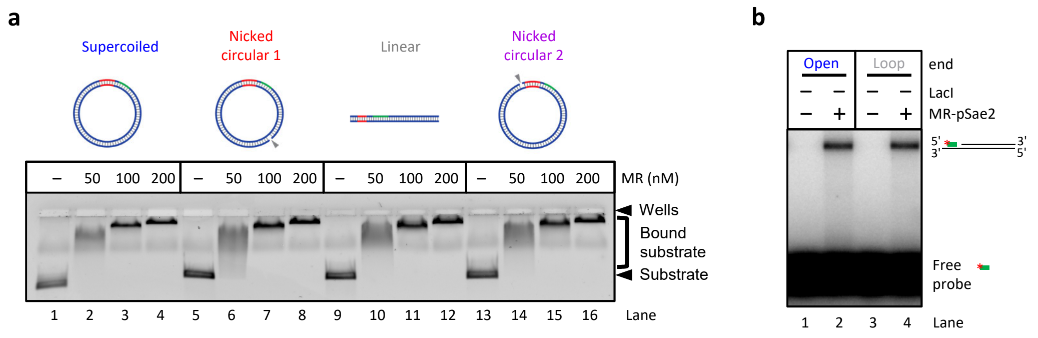

Supplementary Figure 2. A loose DNA end is required for the Mre11 complex activation, related to Figure 2.

a Representative electrophoretic mobility shift assay with MR and the indicated substrates, which were used in Fig. 2b,c. A schematic representation of the substrates is present at the top of the panel. The green line represents the annealing site for the radioactively labeled probe (not used for this experiment) while the LacO site is depicted in red. The position of the nicks is indicated by grey arrowheads. A representative of three independent experiments is shown.

b A representative DNA end resection annealing assay with Mre11-Rad50 (MR) and pSae2, on open and loop ended substrates in the absence of LacI. See Fig. 2d for the cartoon of the DNA substrate used. A representative of two independent experiments is shown.

Source data are provided as a Source Data file.

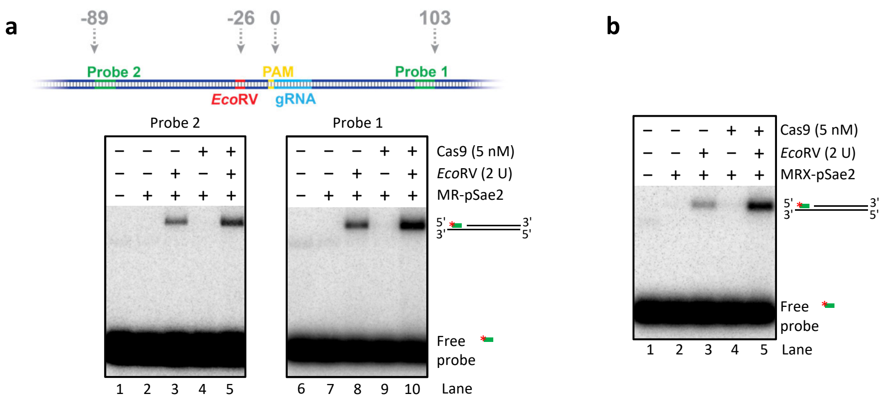

Supplementary Figure 3. Bridging of Cas9-dependent DSBs prevents their immediate processing, related to Figure 3.

a Comparison of DNA end resection on either side of the Cas9 break using a substrate with inverted gRNA site compared to the one used in Fig. 3a. Top, cartoon of the circular plasmid DNA substrate used. The relative distance of the various elements from the boundary between the PAM and the protospacer is indicated in bp. Bottom, representative DNA end resection annealing assays with substrates treated with *Eco*RV and/or Cas9, as indicated. Resection was monitored using a probe on the PAM-proximal (left gel, Probe 2) or PAM-distal (right gel, Probe 1) side of the break. A representative of three independent experiments is shown.

b Representative annealing DNA end resection assay using Mre11-Rad50-Xrs2 (MRX) instead of Mre11-Rad50 (MR), and phosphorylated Sae2 (pSae2). A representative of two independent experiments is shown.

Source data are provided as a Source Data file.

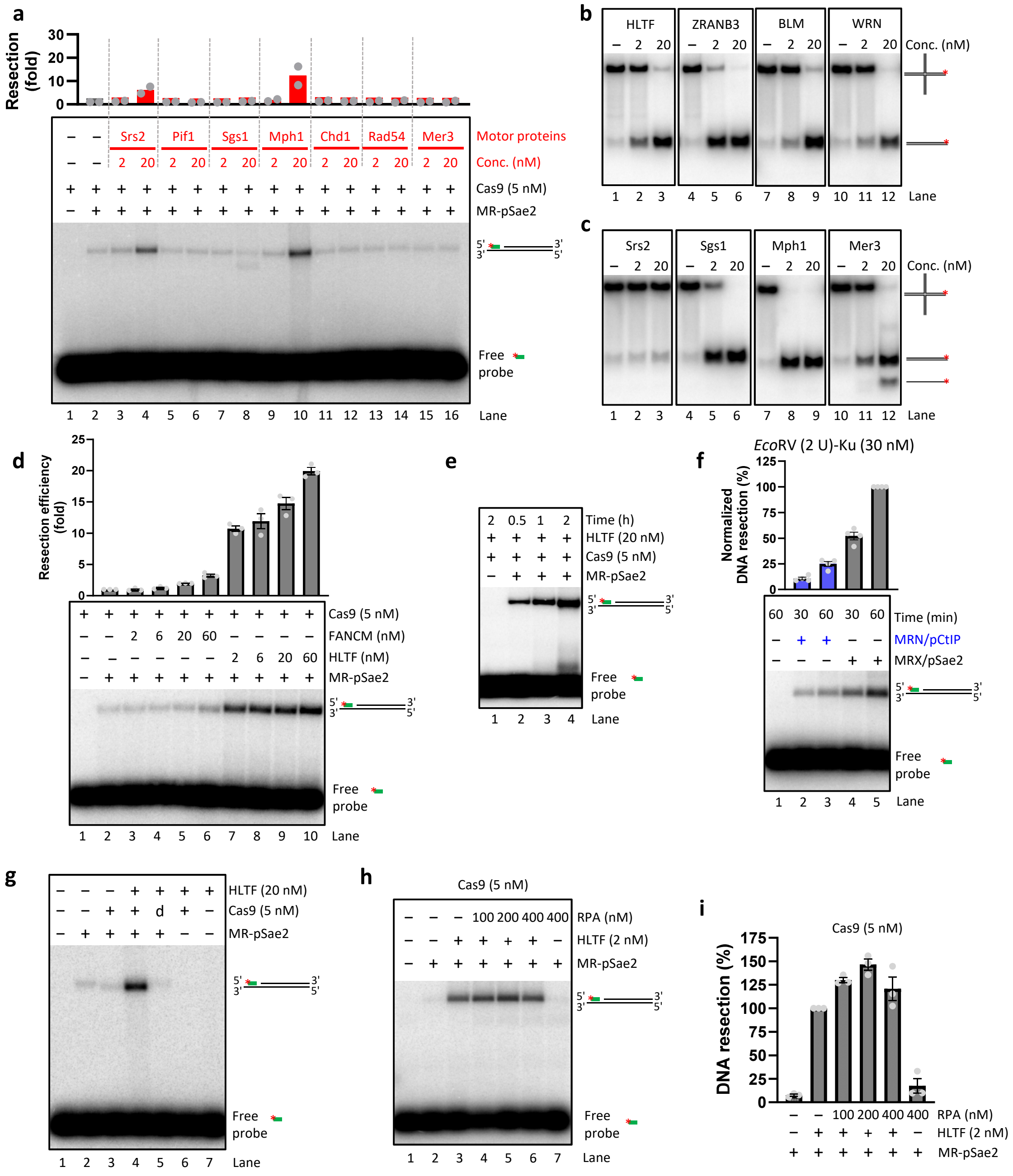

Supplementary Figure 4. HLTF removes Cas9 from DNA post-cleavage complex, related to Figure 4.

Legend in the next page.

Supplementary Figure 4. HLTF removes Cas9 from DNA post-cleavage complex, related to Figure 4.

a Annealing DNA end resection assay of Cas9-mediated DNA breaks by MR and pSae2, in the presence of various yeast DNA translocases. Top, quantitation of resection efficiency expressed as fold increase compared to the “no translocase” sample , which was set to 1. n=2; averages shown. Bottom, a representative of two independent experiment.

b,c Representative DNA branch migration assays using a mobile Holliday junction substrate with selected human motor proteins used in Fig. 4a (b) or yeast motor proteins used in a (c). Right, a cartoon of the substrate and product. The red asterisk indicate the position of the radioactive label. A representative of two independent experiments is shown.

d Annealing DNA end resection assay of Cas9-mediated breaks by MR and pSae2 in the presence of increasing concentration of FANCM or HLTF, as indicated. Top, quantitation of resection efficiency normalized to the resection obtained by MR-pSae2 without translocase, which was set to 1. n=3; error bars, SEM. Bottom, a representative experiment.

e Representative time-course of an annealing DNA end resection assay of Cas9 breaks, catalyzed by MR and pSae2), in the presence of HLTF. A representative of two independent experiments is shown.

f Annealing DNA end resection assay of Ku-blocked EcoRV-mediated DNA breaks by yeast MRXpSae2 or human MRN-pCtIP. Top, quantitation of resection efficiency normalized to the resection obtained by MRX-pSae2 in 1 hour, which was set to 100% . n=4; error bars, SEM. Bottom, a representative experiment.

g Representative annealing DNA end resection assay of DNA substrates incubated with wild type Cas9 (+) or nucleolytically dead Cas9 (d), by MR-pSae2. A representative of two independent experiments is shown.

h Representative annealing DNA end resection assay of Cas9 breaks by MR-pSae2, with HLTF, in the presence of increasing concentrations of human RPA, as indicated.

i Quantitation of experiments such as shown in h. n=3; error bars, SEM. DNA resection efficiency was normalized to the sample without RPA, which was set to 100 %.

Source data are provided as a Source Data file.

a

b

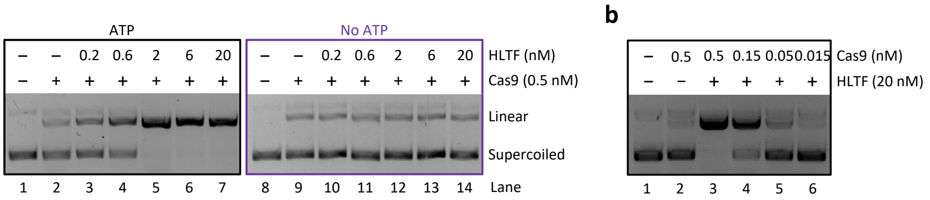

c **DNA substrate for 2-color experiments**

```
TS:   5'-AGCAAGCTGACGTTTGTACTCCAGCGTCTCATCTTTATACATCAGCAGAGATTTCTGCTGTGC-AGACAGCTGACGACGTTGCG-3'
NTS:  3'-TCGTTCGACTGCAAACATGAGGTCGCAGAGTAGAAATATGTAGTCGTCTCTAAAGACGACACG TCTGTCGACTGCTGCAACGCAAB-5'
```

GGT represents the PAM sequence on the NTS.
T represents modified thymine /iAmMC6T/ with Cy5 labeling.
The dash (–) indicates the linkage opposite to the nick

Bold B indicates the position of the 5'-biotin
The sequence of the guide RNA is underlined

d

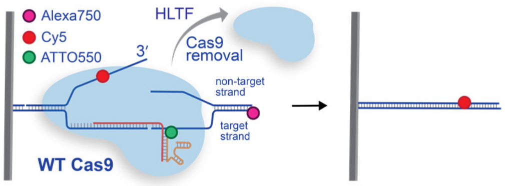

$$E21 = \frac{ATTO550}{Cy5 + ATTO550}$$

$$E23 = \frac{Alexa750}{Cy5 + Alexa750}$$

e **DNA substrate for 3-color experiments**

```
TS:   5'-Alexa750AGCAAGCTGACGTTTGTACTCCAGCGTCTCATCTTTATACATCAGCAGAGATTTCTGCTGTGC-AGACAGCTGACGACGTTGCG-3'
NTS:            3'-TCGTTCGACTGCAAACATGAGGTCGCAGAGTAGAAATATGTAGTCGTCTCTAAAGACGACACG TCTGTCGACTGCTGCAACGCAAB-5'
```

GGT represents the PAM sequence on the NTS.
T represents modified thymine /iAmMC6T/ with Cy5 labeling.
The dash (–) indicates the linkage opposite to the nick

Bold B indicates the position of the 5'-biotin
The sequence of the guide RNA is underlined

f

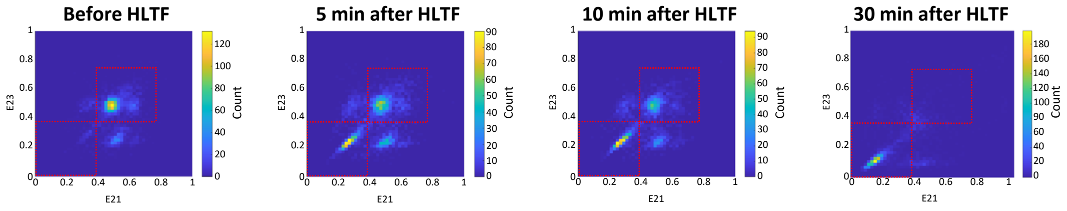

Supplementary Figure 5. HLTF removes Cas9 from DNA post-cleavage complex, related to Figure 5.

a Representative Cas9 multi-turnover assays (three independent experiments) in the presence of 0.5 nM Cas9, 1 nM plasmid DNA and increasing concentrations of HLTF. ATP (1 mM) was included or omitted as indicated.

b Representative Cas9 multi-turnover assay (four independent experiments) in the presence of 20 nM HLTF and decreasing concentrations of Cas9, with 1 nM plasmid DNA.

c A schematic of the substrate used for the single-molecule fluorescence experiments in Fig. 4i-k. The position of the different elements and fluorophores is presented. TS: target strand; NTS: non-target strand.

d A cartoon of the single-molecule fluorescence-based Cas9 dissociation assay using 3 fluorophores. The DNA is visualized by Cy5-labeling and Alexa 750 on the cleaved end, while Cas9 is visualized *via* ATTO550 labeling of the tracrRNA. The formulas used for the calculation of the signal intensity ratios are presented on the right. The E21 ratio was used as a measure of Cas9 binding to the DNA while E23 was used as a measure of whether the two DNA ends were bridged.

e A schematic of the substrate used for the single-molecule fluorescence experiments in f. The position of the different elements and fluorophores is presented.

f A representative time-course experiment of the removal of WT Cas9 by HLTF. Red dashed squares indicate the free DNA (bottom-left) and post-cleavage complex (top-right) for easier visualization. A representative of two independent experiments is shown.

Source data are provided as a Source Data file.

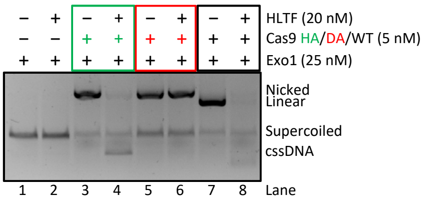

Supplementary Figure 6. HLTF acts on 3' ends released by Cas9 after DNA cleavage, related to Figure 6.

Representative Exo1-mediated resection of plasmid-based DNA substrate cleaved with Cas9 HA, DA or WT variants and its dependence on HLTF. cssDNA: circular single-stranded DNA. Reactions were carried out in the presence of 237 nM yeast RPA. A representative of three independent experiments is shown.

Source data are provided as a Source Data file.

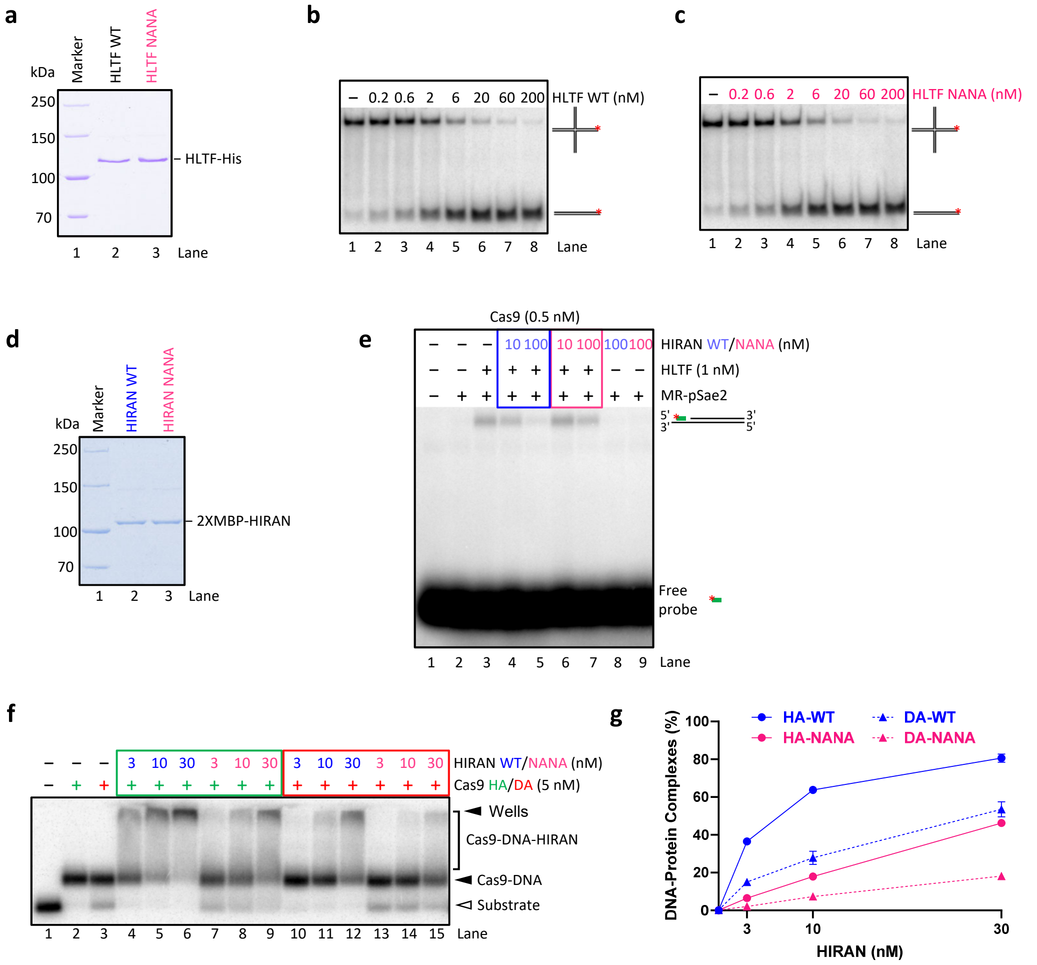

Supplementary Figure 7. The HIRAN domain of HLTF is important for Cas9 removal, related to Figure 7.

a Coomassie-stained gel of HLTF WT and the NANA mutant.

b Representative DNA branch migration assay (four independent experiments) of a Holliday junction substrate with HLTF WT. A cartoon of the substrate and product is presented on the right of the gel. The red asterisks indicate the position of the radioactive label.

c Representative DNA branch migration assay (three independent experiments) of a Holliday junction substrate with the HLTF NANA mutant. A cartoon of the substrate and product is presented on the right of the gel. The red asterisks indicate the position of the radioactive label.

d Coomassie-stained gel of purified WT and NANA mutant of the HIRAN domain of HLTF.

e Representative annealing DNA end resection assay (three independent experiments) by MR-pSae2 in the presence of 0.5 nM Cas9, 1 nM HLTF and increasing concentrations of the HIRAN domain (WT or NANA) variant of HLTF.

f Representative *in vitro* DNA binding assay of 2xMBP-HIRAN (HIRAN) WT or NANA variants on oligo-based DNA substrate reacted with Cas9 HA or DA nickases. The substrate was incubated with the indicated Cas9 variant for 30 minutes at 37 °C, the indicated amount of HIRAN domain was added and further incubated for 10 minutes at 37 °C. The DNA-Protein complexes were then separated on a 1% TAE agarose gel at 4 °C.

g Quantitation of DNA-Cas9-HIRAN complexes formation in experiments such as shown in f. n=3; error bars, SEM.

Source data are provided as a Source Data file.

Supplementary Table 1. Oligonucleotides used in this study.

Oligonucleotides used for the construction of protein expression vectors, plasmid- and oligonucleotide-based substrates and probes for the annealing DNA end resection assay.

Name	Sequence (5' to 3')
LacI-for	CAGACATATGAAACCAGTAACGTTATACG
LacI_6xHis-rev	TCTGAAGCTTAATGGTGATGGTGATGGTGGCTACCGCGGGGAGAGGCGGTTTG
FlagDNL4FO	GCGCGCGGATCCATGGACTACAAAGACGATGACGACAAGATGATATCAGCACTAGATTC
FlagDNL4RE	CGCGCGCCCGGGTCAGTAGTTGACTACGGGGAAGTC
LIF1FO	GCGCGCGCTAGCATGTCCCAGCTGACGGAGTTC
LIF1RE	CGCGCGCTCGAGCTATGTTTCTATATCCGTTTCC
NheI-hHELQ-FP	CCTGCCGCTAGCATGGATGAATGTGGTTCCCGCATCCG
XmaI-hHELQ-RP	CCTGCCCCCGGGCGCTTTGTCAGTGGAAGAAGCCACAGC
FANCJ_to_pFB_for	GATCGGTCTCAGATCGCGGCCGCGCCACCATG
FANCJ_to_pFB_rev	GATCGAAGACTAAGCTGGTCTCTAGCTTTATTTGTCATCGTCGTCCTTGTAG
HLTF_NANA_for	CTGCAGCGAGATCCTGCTGCCCCCTACGACAAGAAC
HLTF_NANA_rev	GTTCTTGTCGTAGGGGGCAGCAGGATCTCGCTGCAG
HLTF_1-180_for	CCCGCTAGCATGAGCTGGATGTTC
HLTF_1-180_rev	TGGTGCTCGAGCCCGGGGAATCCCAGGGTCTTTGGAGCTG
CD.Cas9.GJXV6830.AD	/AlTR1/rUrArCrUrCrGrGrGrArArArCrArUrArUrGrArArUrGrUrUrUrArGrArGrCrUrArUrGrCrU/AlTR2/
pUC19_Cas12a_1	/AlTR1/rUrArArUrUrUrCrUrArCrUrCrUrUrGrUrArGrArUrCrGrArUrUrCrArUrArUrGrUrUrUrCrCrCrGrArGrUrA/AlTR2/
Even_A	CATGTGATATCGAAGACGCTGAGA
Even_B	AGCTTCTCAGCGTCTTCGATATCA
Even(WT)_1	TGAGGAATTGTGAGCGGATAACAATTGC
Even(WT)_2	CTCAGCAATTGTTATCCGCTCACAATTC
Hairpin_integrase_LacO	GATGCATGAGGTGGAGTACGCGCCCGGGGAGCCCAAGGGCACGCCCTGGCACCCGCACCGCGGCAATTGTTATCCGCTCACAATTACGCGAAGCGTAATTGTGAGCGGATAACAATTGCCGCGGTGCGGGTGCCAGGGCGTGCCCTTGGGCTCCCCGGGCGCGTACTCCACCTCATGCATC
Open_int_LacO_TOP	GCGTAATTGTGAGCGGATAACAATTGCCGCGGTGCGGGTGCCAGGGCGTGCCCTTGGGCTCCCCGGGCGCGTACTCCACCTCATGCATC
Open_int_LacO_BOT	GATGCATGAGGTGGAGTACGCGCCCGGGGAGCCCAAGGGCACGCCCTGGCACCCGCACCGCGGCAATTGTTATCCGCTCACAATTACGC
pUC19_0xLacO_Cas_FOR	GAAACATATGAATCGGAAAGAGTTGACACCAGCTGC
pUC19_0xLacO_Cas_REV	GCAGCTGGTGTCAACTCTTTCCGATTCATATGTTTC

pUC19_0xLacO_Cas_rev_oligo1	AGCTTTTCCGATTCATATGTTTCCCGAGTAGAGTTGACACCAGCTGCA
pUC19_0xLacO_Cas_rev_oligo2	GCTGGTGTCAACTCTACTCGGGAAACATATGAATCGGAAA
pUC19_0xLacO_Cas_1kb_for	TGTAGCACCGCCTACATACC
pUC19_0xLacO_Cas_1kb_rev	ATTATCATGACATTAACC
Cas9_target1_FO[a]	CG**CATAACTCAATTTGTAAAAAA**TGGTATTGAGTCGCTCAAGTATTCAAAGCAACTATAGCAAACTCGTGATAGCTTGTCA
Cas9_target1_RE	TGACAAGCTATCACGAGTTTGCTATAGTTGCTTTGAATACTTGAGCGACTCAATACCATTTTTTACAAATTGAGTTATGCG
Cas9-TOP[a]	GTAAGTGCCGCTGAGAAGCT**TACTCGGGAAACATATGAAT**CGGAAAGAGTTCCCCGGGCGCGTACTCCACCTCATGCATC
Cas9-BOT	GATGCATGAGGTGGAGTACGCGCCCGGGGAACTCTTTCCGATTCATATGTTTCCCGAGTAAGCTTCTCAGCGGCACTTAC
XO1	GACGCTGCCGAATTCTACCAGTGCCTTGCTAGGACATcTTTGCCCACCTGCAGGTTCACCC
XO2	TGGGTAAACCTGCAGGTGGGCAAAGATGTCCATCTGTTGTAATCGTCAAGCTTTATGCCGTT
XO1c.MM2	GGGTGAACCTGCAGGTGGGCAAAAATGTCCTAGCAAGGCACTGGTAGAATTCGGCAGCGTC
XO2c.MM	GAACGGCATAAAGCTTGACGATTACAACAGATGGACATTTTTGCCCACCTGCAGGTTCACCC
Probe 1[b]	GGGACTGGGAAAACCCTGGCGT
Probe 2[b]	GGAAAGGCCGCGTTGCTGGCGT
TS	AGCAAGCTGACGTTTGTACTCCAGCGTCTCATCTTTATACATCAGCAGAGATTTCTGCTGTGCAGACAGCTGACGACGTTGCG
NTS[b]	GCACAGCAGAAATCTCTGCTGATG**/Cy5-labeled T/**ATAAAGATGAGACGCTGGAGTACAAACGTCAGCTTGCT
22nt-adaptor[c]	**5Biotin**/AACGCAACGTCGTCAGCTGTCT
FRET-crRNA	rGrArUrGrUrArUrArArArGrArUrGrArGrArCrGrCrGrUrUrUrUrArGrArGrCrUrArUrGrCrUrGrUrUrUrUrG
tracrRNA	rGrGrArCrArGrCrArUrArGrCrArArGrUrUrArArArArUrArArGrGrCrUrArGrUrCrCrGrUrUrArUrCrArArCrUrUrGrArArArArArGrUrGrGrCrArCrCrGrArGrUrCrGrGrUrGrCrUrUrUrUrU
TS-1[c]	**Alexa750**/AGCAAGCTGACGTTTGTACTCCAGCGTCTCATCTTTATACATCAGCAGAGATTTCTGCTGTGCAGACAGCTGACGACGTTGCG

[a] The **bold** portion represent the sequence of the crRNA used, while the PAM is underlined.

[b] The underlined sequence represent the sequence that anneals to the substrate DNA.

[c] Modifications such as Alexa750, 5' biotin and Cy5 labeled T are reported in bold.

Supplementary Table 2. Synthetic genes sequence.

Sequence of the synthetic genes for the cloning of expression vectors. All sequences are presented 5' to 3'.

Sequence name: FANCM_N

gaattCGCTAGCATGTCAGGCCGCCAGAGAACCCTGTTTCAAACGTGGGGATCTAGTATAAGCCGAAG
CTCTGGCACGCCGGGCTGCAGTTCCGGGACGGAAAGACCACAGAGTCCTGGATCATCAAAGGCTCCTC
TTCCTGCAGCTGCAGAGGCGCAGTTAGAGTCGGATGATGACGTATTGCTTGTCGCCGCCTATGAGGCC
GAACGGCAATTGTGTTTGGAAAATGGAGGTTTCTGCACCAGTGCCGGGGCACTATGGATCTATCCAAC
CAATTGTCCTGTGAGAGATTATCAGCTCCATATTTCACGCGCGGCTTTGTTCTGTAACACTCTAGTAT
GTTTGCCCACGGGGCTAGGAAAAACTTTTATAGCCGCTGTGGTCATGTACAACTTCTACAGATGGTTC
CCATCCGGCAAAGTGGTTTTTATGGCACCCACAAAGCCACTGGTCACCCAACAGATCGAAGCGTGTTA
TCAGGTTATGGGAATACCACAATCGCACATGGCCGAGATGACCGGTTCTACACAAGCGAGCACGCGCA
AGGAGATATGGTGTTCAAAGCGTGTGTTATTCCTTACCCCCCAGGTAATGGTGAATGATCTGTCGCGT
GGTGCCTGCCCTGCAGCCGAGATTAAATGCTTAGTAATAGACGAAGCCCACAAAGCACTAGGGAACTA
CGCCTACTGTCAGGTGGTCAGGGAGCTGGTAAAATATACAAATCACTTTCGGATTTTAGCACTTTCTG
CCACCCCGGGTTCTGACATAAAGGCGGTGCAGCAGGTAATTACTAATCTGCTGATTGGCCAAATAGAA
CTACGGTCAGAGGACTCACCCGATATCTTAACATACTCCCATGAACGAAAGGTGGAGAAATTAATTGT
GCCGCTTGGGGAAGAATTAGCTGCAATCCAGAAGACATATATTCAGATCCTCGAATCATTCGCTCGCT
CACTAATACAGCGAAATGTATTAATGAGAAGAGACATCCCCAATCTAACGAAGTACCAAATAATACTC
GCCCGGGATCAATTTCGGAAGAATCCCTCGCCAAATATAGTTGGTATACAACAGGGCATCATCGAAGG
GGAATTTGCTATATGTATTTCTCTCTACCACGGGTACGAGCTACTACAACAAATGGGCATGAGATCTC
TGTATTTCTTTCTCTGCGGGATAATGGATGGAACGAAGGGAATGACACGATCTAAAAACGAACTCGGC
AGAAATGAAGACTTCATGAAACTCTACAATCATTTAGAGTGTATGTTCGCCCGTACTAGATCGACTAG
TGCTAATGGAATATCCGCAATTCAGCAAGGTGATAAAAACAAAAAGTTTGTGTATTCCCATCCCAAAC
TGAAAAAGTTAGAGGAAGTTGTGATCGAGCACTTTAAAAGTTGGAACGCTGAGAACACAACCGAGAAA
AAACGTGATGAAACCCGCGTCATGATATTTTCCAGCTTCCGAGATAGCGTCCAAGAAATTGCAGAAAT
GCTAAGTCAACATCAACCTATTATAAGAGTGATGACCTTCGTAGGTCATGCGAGTGGGAAATCAACCA
AGGGATTTACACAAAAGGAGCAATTGGAGGTCGTGAAACAGTTTAGGGACGGGGGCTATAATACCCTG
GTATCCACGTGTGTCGGCGAAGAAGGGCTTGACATCGGGGAGGTTGATCTTATAATCTGTTTTGATAG
TCAGAAATCGCCGATCCGTTTGGTGCAGCGAATGGGACGTACAGGTCGAAAACGCCAAGGTCGTATAG
TCATAATCTTATCAGAAGGTCGTGAAGAGCGCATTTACAATCAGTCACAATCGAATAAACGATCAATT
TACAAGGCGATTAGTTCCAACCGCCAGGTACTCCACTTTTACCAAAGGTCTCCGCGCATGGTTCCTGA
TGGTATAAACCCAAAACTTCATAAAATGTTTATTACGCATGGGGTTTACGAACCGGAGAAGCCTAGCC
GTAACCTGCAGCGTAAGTCAAGCATCTTCTCCTACCGGGACGGTATGAGGCAGTCGTCTCTCAAGAAG
GACTGGTTTCTGTCCGAGGAAGAATTTAAATTATGGAATCGGCTATATAGGCTGCGAGATTCCGATGA
AATTAAAGAAATAACGTTACCCCAAGTCCAATTTTCCTCCCTTCAGAATGAAGAAAACAAACCGGCAC
AGGAGAGCACAACCGGCATCCACCAACTCTCTCTCTCTGAGTGGAGATTGTGGCAGGATCACCCACTA
CCGACTCATCAAGTAGATCACAGTGATCGGTGTAGGCATTTCATCGGGTTGATGCAGATGATTGAAGG
TATGCGTCACGAGGAAGGAGAATGCTCGTACGAGCTAGAGGTCGAGTCATACCTCCAAATGGAGGATG
TAACTAGCACGTTCATCGCGCCGCGGAACGAATCTAACAACTTAGCGTCAGACACTTTCATTACGCAC
AAGAAGTCCTCGTTTATCAAAAACATTAACCAAGGGTCATCCAGCAGCGTGATTGAGTCAGATGAGGA

ATGTGCGGAAATAGTTAAGCAAACTCACATTAAGCCTACCAAGATCGTTTCATTGAAGAAGAAAGTCA
GTAAGGAGATTAAGAAAGACCAATTAAAGAAAGAGAATAATCATGGTATAATTGACAGCGTCGACAAT
GACCGGAACTCCACTGTAGAAAACATATTTCAAGAGGATCTGCCGAATGACAAACGAACAAGTGATAC
GGATGAAATCGCTGCTACGTGCACCATAAACGAAAACGTTATTAAGGAGCCATGTGTCTTGTTGACCG
AATGTCAGTTCACAAATAAATCCACAAGCTCACTTGCCGGAAACGTCTTAGACTCCGGCTATAACTCG
TTCAATGACGAGAAATCTGTCAGTAGTAATCTGTTTCTCCCCTTTGAAGAGGAGTTGTACATTGTAAG
AACTGACGATCAGTTCTATAACTGTCACTCGTTGACGAAGGAGGTTTTAGCCAATGTGGAGCGATTTC
TTTCGTATTCTCCTCCACCCCTGTCCGGTCTGTCTGACCTCGAATATGAGATTGCGAAAGGAACAGCC
CTCGAAAACCTTCTCTTTTTACCTTGTGCCGAACATCTGCGGAGTGATAAATGTACTTGCCTACTAAG
TCATAGTGCGGTTAACTCCCAACAGAATTTGGAGCTGAATAGTCTAAAGTGTATCAACTATCCCTCGG
AAAAGAGCTGCCTGTACGATATCCCTAACGATAACATAAGCGACGAACCCTCCCTATGCGATTGTGAC
GTGCATAAACACAACCAGAATGAGAACCTTGTTCCCAATAACCGGGTTCAGATCCATCGCAGCCCAGC
GCAAAATTTAGTGGGCGAGAACAATCATGATGTGGATAATTCTGATTTACCAGTTCTATCAACTGACC
AGGACGAATCCTTGCTCCTGTTTGAAGATGTAAACACAGAGTTTGATGATGTATCCCTCTCCCCTTTA
AATTCTAAGTCTGAATCCCTGCCGGTCTCGGATAAGACCGCAATATCTGAGACTCCACTTGTGAGTCA
GTTCCTAATATCGGATGAACTCTTGCTCGACAATAACAGCGAACTTCAGGACCAAATAACGAGAGATG
CCAATTCATTCAAGTCCCGAGATCAGCGCGGAGTCCAAGAGGAGAAGGTCAAAAATCATGAGGACATA
TTCGACTGCTCCAGGGATCTGTTTAGCGTTACTTTTGACTTAGGTTTCTGCAGTCCGGATTCCGATGA
CGAGATTCTGGAGCACACATCTGACTCGAACCGCCCCCTTGACGATCTCTATGGCCGATATCTGGAGA
TCAAGGAAATTTCAGATGCGAACTATGTAAGTAATCAGGCGCTGATTCCTCGTGATCATTCCAAGAAC
TTTACATCGGGAACTGTTATCATCCCCTCAAACGAAGATATGCAGAACCCCAATTACGTTCATCTCCC
CCTATCGGCGGCTAAAAATGAAGAGCTGCTCTCACCTGGCTATTCGCAGTTTAGTCTCCCTGTTCAGA
AAAAGGTGATGAGTACTCCACTGAGCAAGTCTAATACCTTAAATTCTTTTAGCAAAATCAGGAAAGAA
ATCCTTAAAACTCCGGACTCATCCAAGGAGAAGGTCAATTTGCAGCGTTTCAAAGAAGCTCTTAATAG
TACGTTCGATTACTCTGAATTTTCTTTGGAAAAAAGCAAGTCATCGGGTCCGATGTACCTTCATAAAA
GCTGCCATTCAGTGGAAGACGGCCAGTTATTGACCTCAAATGAGTCCGAAGATGATGAGATCTTCCGA
CGGAAAGTAAAACGGGCCAAAGGCAACGTCTTAAATAGTCCTGAGGACCAAAAAAATAGCGAGGTCGA
CTCTCCGCTCCACGCAGTTAAAAAACGTAGATTCCCCATCAACCGGAGTGAACTCAGCTCAAGTGACG
AGTCTGAGAACTTTCCTAAACCGTGCTCCCAGCTAGAAGACTTCAAGGTATGTAACGGAAACGCAAGG
AGAGGAATTAAGGTGCCGAAGCGACAATCGCACCTtAAgCACGTTGCTAGAagctt

Sequence name: FANCM_C

---

gaattCGCTAGCACAATCGCACCTtAAgCACGTTGCTAGGAAATTTCTTGATGACGAAGCGGAACTTT
CGGAGGAAGACGCCGAGTACGTTTCTTCAGATGAGAACGACGAGTCTGAGAATGAGCAAGACAGCTCG
CTACTAGACTTCCTAAATGATGAAACACAGCTCTCCCAAGCTATAAATGACAGCGAGATGAGAGCAAT
CTATATGAAAAGTCTAAGGTCCCCAATGATGAACAATAAATATAAGATGATTCATAAAACGCATAAAA
ACATAAATATATTTTCCCAAATTCCAGAGCAAGACGAGACCTATCTTGAAGATTCTTTCTGCGTTGAC
GAAGAAGAGTCTTGCAAAGGTCAATCTAGCGAAGAAGAGGTCTGCGTTGACTTTAACTTAATTACCGA
TGACTGTTTCGCCAACTCGAAAAAATATAAGACGAGGCGGGCTGTTATGTTAAAGGAAATGATGGAGC
AGAATTGCGCGCATAGCAAAAAAAAATTATCTCGTATCATCCTGCCGGACGACTCTAGCGAAGAGGAA
AACAACGTCAACGACAAACGGGAATCCAACATAGCGGTAAATCCAAGTACGGTAAAGAAAAACAAACA
ACAGGATCATTGCTTGAACAGTGTGCCATCGGGGAGTTCCGCACAAAGTAAAGTCCGCTCGACCCCCC
GTGTCAACCCTCTGGCGAAGCAGTCAAAGCAGACCTCATTGAACCTAAAGGATACAATTAGCGAGGTG

TCTGACTTCAAGCCTCAGAATCACAACGAGGTACAGTCTACCACTCCACCGTTTACCACAGTTGACTC
TCAAAAGGACTGCAGGAAGTTCCCTGTTCCCCAAAAAGATGGGAGCGCATTAGAGGATAGCTCGACTT
CGGGAGCATCCTGCAGCAAGTCACGGCCACATTTGGCTGGAACACATACTTCGTTGAGGCTTCCCCAG
GAGGGCAAAGGTACATGTATCTTAGTTGGTGGTCACGAGATCACATCAGGGTTGGAAGTAATATCATC
TTTGAGAGCTATTCACGGGCTGCAAGTAGAAGTGTGTCCGCTGAATGGATGCGATTATATTGTTTCAA
ACAGAATGGTAGTGGAGCGACGCTCGCAGAGTGAAATGTTAAATTCAGTTAATAAAAATAAATTCATC
GAGCAGATCCAGCACCTCCAGAGCATGTTTGAGAGGATATGCGTTATCGTAGAAAAAGACCGTGAAAA
GACTGGTGATACATCCCGCATGTTCAGGCGGACTAAAAGTTACGATTCCCTCTTAACGACCTTAATTG
GCGCGGGGATTCGCATTCTCTTCAGTTCTTGCCAAGAGGAGACGGCGGATCTCCTAAAAGAACTCTCT
CTTGTGGAACAAAGGAAGAATGTTGGCATTCACGTCCCCACTGTCGTAAATTCGAACAAGTCGGAAGC
TCTGCAGTTCTATTTATCGATTCCGAACATATCTTACATTACGGCACTAAATATGTGCCACCAATTTA
GTTCTGTCAAGCGCATGGCTAATTCGTCTCTGCAAGAAATATCTATGTACGCACAAGTAACACACCAA
AAGGCCGAAGAAATCTATAGATACATCCACTACGTATTCGACATCCAAATGCTTCCAAACGACTTGAA
CCAGGACCGTTTAAAGTCGGACATCcatcaccaccatcatcaccatcaccaccacTGAagctt

Sequence name: hRAD54

GCTAGCGAGAACCTGTACTTCCAAGGCTCAATGCGCCGTTCCCTGGCCCCTAGCCAGCTGGCTAAGCG
CAAGCCCGAAGGTCGTTCATGCGACGACGAGGACTGGCAGCCAGGCCTGGTGACTCCTAGGAAGAGAA
AGTCCAGCTCTGAAACCCAGATCCAGGAGTGCTTCCTGTCTCCATTCCGCAAGCCTCTGTCACAGCTG
ACCAACCAGCCTCCCTGCCTGGACTCATCCCAGCACGAAGCCTTCATCCGTAGCATCCTGTCTAAGCC
ATTCAAGGTCCCCATCCCAAACTACCAGGGACCTCTGGGTTCCAGGGCTCTGGGTCTGAAGAGAGCCG
GCGTGAGGAGAGCTCTGCACGACCCACTGGAAAAGGACGCCCTGGTCCTGTACGAGCCACCTCCCCTG
TCAGCTCACGACCAGCTGAAGCTGGACAAGGAGAAGCTGCCCGTGCACGTGGTCGTGGACCCAATCCT
GTCCAAGGTCCTGAGGCCTCACCAGAGAGAAGGCGTGAAGTTCCTGTGGGAGTGCGTGACCTCTCGCC
GTATCCCCGGCAGCCACGGATGCATCATGGCCGACGAAATGGGTCTGGGCAAGACCCTGCAGTGCATC
ACCCTGATGTGGACTCTGCTGCGCCAGTCCCCCGAATGCAAGCCAGAGATCGACAAGGCTGTCGTGGT
CAGCCCAAGCTCTCTGGTGAAGAACTGGTACAACGAGGTCGGAAAGTGGCTGGGTGGCCGTATCCAGC
CTCTGGCCATCGACGGAGGTAGCAAGGACGAAATCGACCAGAAGCTGGAGGGCTTCATGAACCAGCGC
GGAGCTCGTGTGTCATCCCCTATCCTGATCATCTCTTACGAAACTTTCCGCCTGCACGTGGGAGTCCT
GCAGAAGGGTTCAGTGGGCCTGGTCATCTGCGACGAAGGTCACCGTCTGAAGAACTCCGAGAACCAGA
CCTACCAGGCTCTGGACTCTCTGAACACTTCAAGGAGAGTGCTGATCTCCGGTACCCCTATCCAGAAC
GACCTGCTGGAGTACTTCTCCCTGGTGCACTTCGTCAACAGCGGAATCCTGGGTACCGCCCACGAATT
CAAGAAGCACTTCGAGCTGCCTATCCTCAAGGGAAGGGACGCTGCTGCTTCCGAAGCTGACCGTCAGC
TGGGAGAGGAACGCCTGCGTGAGCTGACTTCTATCGTGAACCGTTGCCTGATCCGCCGTACCTCCGAC
ATCCTGAGCAAGTACCTGCCCGTCAAGATCGAACAGGTGGTCTGCTGCCGCCTGACCCCACTGCAGAC
TGAGCTGTACAAGAGGTTCCTGAGACAGGCCAAGCCCGCTGAGGAACTGCTGGAAGGAAAGATGTCTG
TGAGCTCTCTGTCATCCATCACTTCACTGAAGAAGCTGTGCAACCACCCAGCCCTGATCTACGACAAG
TGCGTGGAGGAAGAGGACGGCTTCGTCGGAGCCCTGGACCTGTTCCCACCTGGATACAGCTCTAAGGC
TCTGGAGCCTCAGCTGTCCGGCAAGATGCTGGTGCTGGACTACATCCTGGCTGTCACTAGGTCCAGAT
CATCCGACAAGGTGGTCCTGGTGAGCAACTACACCCAGACTCTGGACCTGTTCGAAAAGCTGTGCAGG
GCCAGGAGATACCTGTACGTCAGACTGGACGGTACCATGTCCATCAAGAAGCGCGCTAAGGTGGTCGA
GCGTTTCAACAGCCCTAGCTCTCCCGACTTCGTGTTCATGCTGTCATCCAAGGCCGGCGGATGCGGAC
TGAACCTGATCGGTGCTAACCGCCTGGTCATGTTCGACCCTGACTGGAACCCTGCTAACGACGAACAA

GCTATGGCTCGCGTCTGGAGGGACGGTCAGAAGAAGACTTGCTACATCTACAGGCTGCTGTCTGCCGG
CACCATCGAAGAGAAGATCTTCCAGAGACAGTCACACAAGAAGGCTCTGAGCTCTTGCGTGGTCGACG
AAGAGCAGGACGTGGAAAGGCACTTCAGCCTGGGTGAACTGAAGGAGCTGTTCATCCTGGACGAGGCT
TCACTGTCTGACACTCACGACAGGCTGCACTGCCGCCGTTGCGTCAACTCTAGGCAGATCAGACCCCC
ACCTGACGGTTCTGACTGCACTTCAGACCTGGCCGGCTGGAACCACTGCACCGACAAGTGGGGACTGA
GGGACGAAGTGCTGCAAGCTGCTTGGGACGCTGCTTCTACCGCTATCACTTTCGTCTTCCACCAGAGA
TCACACGAAGAGCAGCGCGGCCTGCGTGACTACAAGGACGACGACGACAAGTAACCCGGGATCGGATC
CCGGGCCCGTCGACTGCAGAGGCCTGCATGCAAGCTT

Sequence name: hPIF1

GCTAGCGGATCCATGCTGAGCGGAATCGAAGCTGCCGCTGGCGAGTACGAAGACTCTGAGCTGCGTTG
CAGGGTCGCTGTGGAAGAACTGTCACCTGGTGGCCAGCCACGCCGTAGGCAGGCTCTGCGTACCGCCG
AACTGTCCCTGGGCAGGAACGAGAGACGCGAACTGATGCTCAGGCTGCAAGCTCCAGGCCCTGCTGGA
AGACCAAGGTGCTTCCCTCTGCGTGCCGCTAGGCTGTTCACTAGGTTCGCTGAGGCCGGACGTTCTAC
CCTGAGGCTGCCTGCTCACGACACTCCCGGAGCTGGTGCTGTGCAGCTGCTGCTGTCAGACTGCCCTC
CCGACAGACTGCGTAGGTTCCTGAGAACCCTGAGGCTGAAGCTGGCCGCTGCCCCCGGCCCAGGACCT
GCTTCCGCCCGCGCTCAGCTGCTGGGTCCAAGGCCAAGGGACTTCGTCACCATCAGCCCAGTGCAGCC
TGAGGAAAGACGCCTCAGAGCTGCTACTCGCGTCCCAGACACCACTCTGGTGAAGAGGCCTGTGGAAC
CACAAGCTGGAGCTGAGCCATCCACTGAAGCTCCTAGATGGCCTCTGCCCGTGAAGCGCCTGTCTCTG
CCCTCAACTAAGCCACAGCTGAGCGAGGAACAGGCTGCCGTCCTGCGTGCTGTGCTGAAGGGACAGTC
CATCTTCTTCACCGGCAGCGCCGGTACTGGCAAGTCTTACCTGCTGAAGCGCATCCTGGGAAGCCTGC
CACCTACCGGTACTGTCGCCACCGCTTCTACTGGCGTGGCTGCCTGCCACATCGGAGGTACCACTCTG
CACGCCTTCGCTGGAATCGGTTCTGGTCAAGCTCCTCTGGCCCAGTGCGTGGCCCTGGCTCAACGTCC
CGGCGTGAGGCAGGGATGGCTGAACTGCCAGCGCCTGGTCATCGACGAGATCTCAATGGTGGAAGCTG
ACCTGTTCGACAAGCTGGAGGCTGTGGCTCGTGCCGTGAGGCAGCAGAACAAGCCATTCGGCGGAATC
CAGCTGATCATCTGCGGAGACTTCCTGCAGCTGCCCCCAGTCACCAAGGGTTCCCAGCCTCCCAGATT
CTGCTTCCAGTCCAAGAGCTGGAAGCGCTGCGTCCCTGTGACCCTGGAACTGACTAAAGTGTGGCGCC
AGGCTGACCAGACTTTCATCTCACTGCTGCAGGCCGTCAGACTGGGAAGGTGCTCTGACGAGGTGACC
AGGCAGCTGCAGGCTACTGCCAGCCACAAGGTCGGTCGTGACGGCATCGTGGCTACCAGGCTGTGCAC
TCACCAGGACGACGTCGCCCTGACCAACGAGCGTAGGCTGCAGGAACTGCCTGGAAAGGTGCACCGCT
TCGAAGCTATGGACTCAAACCCAGAGCTGGCTTCCACTCTGGACGCCCAGTGCCCTGTGTCTCAGCTG
CTGCAGCTGAAGCTGGGTGCCCAGGTCATGCTGGTGAAGAACCTGTCTGTCTCAAGAGGCCTGGTGAA
CGGAGCTCGCGGTGTGGTCGTGGGATTCGAGGCCGAAGGACGTGGTCTGCCTCAGGTCAGGTTCCTGT
GCGGAGTCACCGAAGTGATCCACGCTGACCGTTGGACTGTGCAGGCTACTGGTGGCCAACTGCTGTCT
CGTCAACAGCTGCCACTGCAGCTGGCTTGGGCCATGTCCATCCACAAGAGCCAGGGTATGACTCTGGA
CTGCGTCGAGATCAGCCTGGGCAGAGTGTTCGCTTCTGGACAGGCTTACGTCGCCCTGTCTAGAGCCC
GCTCACTGCAGGGTCTGAGGGTGCTGGACTTCGACCCCATGGCTGTCAGATGCGACCCACGCGTGCTG
CACTTCTACGCTACTCTCAGACGCGGACGTTCCCTGAGCCTGGAGTCACCAGACGACGACGAAGCTGC
CTCCGACCAGGAGAACATGGACCCAATCCTGCACCATCATCACCATCATCACCACCACCACTGAAGCT
TATCGGATCCCGGG
