# Supplementary material for: HLTF disrupts Cas9-DNA post-cleavage complexes to allow DNA break processing
Source: Nat Commun. 2024 Jul 10;15:5789. doi: 10.1038/s41467-024-50080-y (PMC11237066; doi:10.1038/s41467-024-50080-y)
Supplement: Supplementary file 3 — Reporting Summary [file 41467_2024_50080_MOESM3_ESM.pdf]

nature portfolio

Corresponding author(s): Petr Cejka

Last updated by author(s): Jun 14, 2024

Reporting Summary

Nature Portfolio wishes to improve the reproducibility of the work that we publish. This form provides structure for consistency and transparency in reporting. For further information on Nature Portfolio policies, see our Editorial Policies and the Editorial Policy Checklist.

Statistics

For all statistical analyses, confirm that the following items are present in the figure legend, table legend, main text, or Methods section.

n/a	Confirmed	
☐	☒	The exact sample size (*n*) for each experimental group/condition, given as a discrete number and unit of measurement
☐	☒	A statement on whether measurements were taken from distinct samples or whether the same sample was measured repeatedly
☐	☒	The statistical test(s) used AND whether they are one- or two-sided<br>*Only common tests should be described solely by name; describe more complex techniques in the Methods section.*
☒	☐	A description of all covariates tested
☒	☐	A description of any assumptions or corrections, such as tests of normality and adjustment for multiple comparisons
☐	☒	A full description of the statistical parameters including central tendency (e.g. means) or other basic estimates (e.g. regression coefficient) AND variation (e.g. standard deviation) or associated estimates of uncertainty (e.g. confidence intervals)
☐	☒	For null hypothesis testing, the test statistic (e.g. *F*, *t*, *r*) with confidence intervals, effect sizes, degrees of freedom and *P* value noted<br>*Give P values as exact values whenever suitable.*
☒	☐	For Bayesian analysis, information on the choice of priors and Markov chain Monte Carlo settings
☒	☐	For hierarchical and complex designs, identification of the appropriate level for tests and full reporting of outcomes
☒	☐	Estimates of effect sizes (e.g. Cohen's *d*, Pearson's *r*), indicating how they were calculated

Our web collection on statistics for biologists contains articles on many of the points above.

Software and code

Policy information about availability of computer code

Data collection	Gels were acquired using a Typhoon Phosphor Imager FLA 9500 (version 1.0), Quantum CX5 Edge 18.06 and photo scanner operated with CanoScan 9000F Mark II scanner and ImageCapture v6.6(525) software. Blots were captured using a Li-Cor Odyssey CLx imaging system.
Data analysis	Gels were analyzed using ImageJ 1.53g and the results were plotted using Graph Pad Prism 10.2.2 (397).

For manuscripts utilizing custom algorithms or software that are central to the research but not yet described in published literature, software must be made available to editors and reviewers. We strongly encourage code deposition in a community repository (e.g. GitHub). See the Nature Portfolio guidelines for submitting code & software for further information.

Data

Policy information about availability of data

All manuscripts must include a data availability statement. This statement should provide the following information, where applicable:

- Accession codes, unique identifiers, or web links for publicly available datasets
- A description of any restrictions on data availability
- For clinical datasets or third party data, please ensure that the statement adheres to our policy

No large datasets were generated or analyzed during the current study. All data supporting the findings of this study are available within the paper and its supplementary information files.

Research involving human participants, their data, or biological material

Policy information about studies with human participants or human data. See also policy information about sex, gender (identity/presentation), and sexual orientation and race, ethnicity and racism.

Reporting on sex and gender	N/A
Reporting on race, ethnicity, or other socially relevant groupings	N/A
Population characteristics	N/A
Recruitment	N/A
Ethics oversight	N/A

Note that full information on the approval of the study protocol must also be provided in the manuscript.

Field-specific reporting

Please select the one below that is the best fit for your research. If you are not sure, read the appropriate sections before making your selection.

☒ Life sciences ☐ Behavioural & social sciences ☐ Ecological, evolutionary & environmental sciences

For a reference copy of the document with all sections, see nature.com/documents/nr-reporting-summary-flat.pdf

Life sciences study design

All studies must disclose on these points even when the disclosure is negative.

Sample size	The number of repeats was chosen based on what is common and/or practical in the field.
Data exclusions	Data points were excluded only when there was a valid reason to do so, such as errors during the assay execution and/or imaging, or failure of experimental apparatuses (e.g. ruined gel wells).
Replication	The experiments were repeated multiple times, as indicated in the figure legend.
Randomization	Randomization is not required for the experiments presented in this study as the results are quantifiable and, therefore, there is no risk of introducing a bias.
Blinding	Blinding was prevented by the loading order of the sample. This was nevertheless not a probem as quantification was carried out objectively with a dedicated software.

Reporting for specific materials, systems and methods

We require information from authors about some types of materials, experimental systems and methods used in many studies. Here, indicate whether each material, system or method listed is relevant to your study. If you are not sure if a list item applies to your research, read the appropriate section before selecting a response.

Materials & experimental systems

n/a	Involved in the study	
☐	☒	Antibodies
☐	☒	Eukaryotic cell lines
☒	☐	Palaeontology and archaeology
☒	☐	Animals and other organisms
☒	☐	Clinical data
☒	☐	Dual use research of concern
☒	☐	Plants

Methods

n/a	Involved in the study	
☒	☐	ChIP-seq
☒	☐	Flow cytometry
☒	☐	MRI-based neuroimaging

Antibodies

Antibodies used	The antibodies used were as follows: anti-Cas9 (1:1,000, Novus Biologicals, NBP280679), anti-HLTF (1:500, Abcam, ab17984), anti-γH2AX (1:5,000, pSer 139, Millipore γH2AX (1:5,000, pSer 139, Millipore 05-636), anti-pRPA S4/S8 (1:1,000, Bethyl Laboratories, Cat#

	A300-245A), anti-Tubulin (1:5,000, Sigma, #T-5168), fluorescent secondary antibody anti-mouse IRDye 800CW and anti-rabbit 680RD (LI-COR Biosciences).
Validation	The antibodies used in this study are commercially available and application data are available on the relevant websites.

Eukaryotic cell lines

Policy information about cell lines and Sex and Gender in Research

Cell line source(s)	We used Sf9 cells adapted for growth in suspension, which are available from the cell line collection of the Institute of Molecular Research University of Zurich. MCF10A cells were obtained from ATCC.
Authentication	The cell lines were not authenticated.
Mycoplasma contamination	The cell lines were not tested for mycoplasma contamination.
Commonly misidentified lines (See ICLAC register)	No misidentified cell lines were used in this study.

Plants

Seed stocks	N/A
Novel plant genotypes	N/A
Authentication	N/A
